# Mixed-Matrix Membranes Comprising of Polysulfone and Porous UiO-66, Zeolite 4A, and Their Combination: Preparation, Removal of Humic Acid, and Antifouling Properties

**DOI:** 10.3390/membranes10120393

**Published:** 2020-12-04

**Authors:** Tanzila Anjum, Rahma Tamime, Asim Laeeq Khan

**Affiliations:** 1 Department of Environmental Sciences and Policy, Lahore School of Economics, Lahore 54000, Pakistan; tanzila.anjum@gmail.com; 2 Department of Chemical Engineering, COMSATS University Islamabad, Lahore 54000, Pakistan

**Keywords:** mixed-matrix membranes, UiO-66, Zeolite 4A, antifouling

## Abstract

High-performance Mixed-Matrix Membranes (MMMs) comprising of two kinds of porous fillers UiO-66 and Zeolite 4Aand their combination were fabricated with polysulfone (PSf) polymer matrix. For the very first time, UiO-66 and Zeolite 4A were jointly used as nanofillers in MMMs with the objective of complimenting synergistic effects. The individual and complimentary effects of nanofillers were investigated on membrane morphology and performance, pure water flux, humic acid rejection, static humic acid adsorption, and antifouling properties of membranes. Scanning Electron Microscopy (SEM) analysis of membranes confirmed that all MMMs possessed wider macrovoids with higher nanofiller loadings than neat PSf membranes and the MMMs (PSf/UiO-66 and PSf/Zeolite 4A-UiO-66) showed tendency of agglomeration with high nanofiller loadings (1 wt% and 2 wt%). All MMMs exhibited better hydrophilicity and lower static humic acid adsorption than neat PSf membranes. Pure water flux of MMMs was higher than neat PSf membranes but the tradeoff between permeability and selectivity was witnessed in the MMMs with single nanofiller. However, MMMs with combined nanofillers (PSf/Zeolite 4A-UiO-66) showed no such tradeoff, and an increase in both permeability and selectivity was achieved. All MMMs with lower nanofiller loadings (0.5 wt% and 1 wt%) showed improved flux recovery. PSf/Zeolite 4A-UiO-66 (0.5 wt%) membranes showed the superior antifouling properties without sacrificing permeability and selectivity.

## 1. Introduction

Polymeric filtration membranes have been used extensively in water treatment because of their high separation efficiency, easy automation, compactness of design, and relatively low energy consumption [1]. They have been used in a wide range of applications such as the removal of turbidity, hardness, organic matters, microorganisms, micro-pollutants, dyes, as well as for the fractions of dissolved salts from natural and wastewater, and sea water desalination [2]. The structure and performance of these membranes is shown to be highly affected by the physical and chemical properties of the selected polymer [3].

Various polymers such as Polyethersulfone (PES), Polysulfone (PSf), Polyvinylidene fluoride (PVDF), Cellulose Acetate (CA), Polyacrylonitrile (PAN) and Polyimide (PI) have been used to prepare membranes with desired properties [4]. Among these polymers, PSf has been used in drinking water production due to its distinctive physicochemical properties such as high thermal stability (150–170 °C), suitable mechanical strength, wide ranges of pore size, high chemical resistance over entire range of pH, and an outstanding film forming ability [5]. However, PSf is generally associated with an undesirable hydrophobicity, which makes it prone to severe reversible and/or irreversible organic fouling resulting from the attachment of organic foulants on the membrane surface and/or their accumulation inside the membrane structure [6]. Such fouling causes several inefficiencies in membrane performance such as decreased permeation flux, reduced membrane life, change in selectivity and rejection, and high operational energy and maintenance [7]. This implies that the effectiveness of filtration membranes mainly depends on their ability to control the membrane fouling. However, fouling control is very challenging due to complexity of the phenomenon that results from the combination of factors such as properties of foulant (concentration, hydrophobicity, molecular weight, molecular size, diffusion, solubility, charge), characteristics of membrane (molecular weight cut-off, porous structure, surface roughness, hydrophobicity, and surface charge), and hydrodynamic conditions (permeate flux, flow velocity and solution temperature) [8].

There has been several attempts to restrict fouling involving methods such as pretreatment (coagulation, adsorption, oxidation and biological treatment) [9] and modification in operational conditions (running modes, rinsing, chemical cleaning, air scouring) [10]. However, a general conclusion is that to improve the resistance against fouling without compromising efficiency, the surface hydrophilicity of membranes should be enhanced [11]. To this end, various approaches have been adopted to increase the surface hydrophilicity of filtration membranes including the development of composite membranes via interfacial polymerization [12], surface grafting [13], surface coating [14], electron beam irradiation [15] and addition of nanofillers in membrane–polymer matrix [16]. Among these methods, preparation of MMMs by blending hydrophilic nanofillers in hydrophobic polymer matrix has attracted considerable attention because of their easy preparation with comparable reduction in membrane fouling [17]. Moreover, MMMs can be scaled up easily and have long-term operational stability with excellent separation performance as compared to polymer membranes [18].

Nanofillers are shown to increase membrane performance in terms of its permeability [19], rejection [20] and antifouling properties [21]. Several types of nanofillers such as titanium oxide (TiO_2_) [22], carbon nano-tubes [23,24], alumina (Al_2_O_3_) [25], zeolites [26], zinc oxide (ZnO) [27,28], MOFs [29,30], silica (SiO_2_) [31,32], and graphene oxide (GO) [33] have been used to enhance the antifouling properties of filtration membranes. These nanofillers have useful effects on membrane structure and characteristics. They often enhance the antifouling properties of membranes by increasing their surface hydrophilicity. Some nanofillers (such as titanium oxide, carbon nano-tubes, zinc oxide, silica etc.) provide the sites of carboxylic and hydroxyl groups, which improve hydrogen bonding with water molecules and lead to the development of a water layer on membrane surface. This water layer inhibits the foulant material to deposit on the membrane surface and leads to reduced fouling [34]. Membranes could also result in undesirable properties such as agglomeration, poor dispersion [35], low stability during operation, tradeoff between permeability and selectivity [36], poor compatibility between polymer matrix and nanofillers, and improved fouling resistance while sacrificing permeability or selectivity [37]. 

To mitigate the undesirable properties of MMMs, a recent approach proposes combining two fillers, and the existing studies showed some promising results. For example, Zeng et al., [38] prepared the TiO_2_-HNTs/PVDF ultrafiltration membranes and reported not only significantly improved permeability, hydrophilicity, porosity and antifouling performance of membranes but also enhanced dispersion of nanofiller in polymer matrix as compared to neat PVDF membrane and TiO_2_/PVDF membrane. In another study, Chung et al. [39] casted PSf composite membranes by functionalizing ZnO on GO for better dispersion of nanofiller. They also reported that PSf/ZnO/GO membranes showed remarkably improved hydrophilicity, permeability, humic acid rejection, antifouling properties, and bacterial control along with better dispersion of nanofiller. Similarly, Ma et al., [40] used UiO-66 Metal-Organic Framework (MOF) with combination of GO nanosheets and investigated the antifouling behavior of composite membranes. They found that UiO-66 successfully inhibited the stacking of GO sheets and the composite membranes (PES/UiO-66-GO) showed impressive antifouling performance, higher hydrophilicity, better permeability and improved rejection of dyes as compared to PES and GO/PES membranes.

In this study, we contribute to this emerging literature by combining two porous fillers Zeolite 4A and UiO-66in PSf matrix with an objective to exploit their positive properties and investigate their effects on membrane morphology and its performance, such as hydrophilicity, pure water flux, humic acid rejection, and antifouling properties. MOFs are crystalline substances which consist of metal ions coordinated with organic linker. They possess large surface areas, wide range of functionality, flexibility in chemical composition and good compatibility with polymers due to their organic linker [41]. This compatibility makes MOFs suitable to achieve proper interface morphology, and thus, improved separation performance [42].

Among these MOFs, UiO-66 [Zr_6_O_4_(OH)_4_(O_2_C-C_6_H_4_-CO_2_)_6_] is made of Zr_6_O_4_(OH)_4_ clusters that are linked with 12 terephthalate ligands. Its 3D network consists of octahedral and tetrahedral cages that are interconnected by triangular windows. Its diameter is 6 Å which is greater than the size of water molecules (~2.8 Å) and lesser than most of the organic contaminants (larger than 6.0 Å) [43]. The outstanding chemical, thermal stability and hydrophilicity of UiO-66 make it appropriate to incorporate in PSf polymer for water treatment [44]. 

Zeolites are crystalline alumino-silicates which are considered very promising nanofillers due to their superior hydrophiliciy. They have crystalline cage-like structure, precise nano-channel, and negative charges which make them suitable as catalysts, ion exchangers, and absorbents. Zeolites have suitable interfacial contact with glassy polymers because of their ordered meso-porosity and external surface area [45]. Among the zeolites, Zeolite 4A (Na_12_ [(AlO_2_)_12_(SiO_2_)_12_] 27H_2_O) has high hydrophilicity due to high aluminum content (Si/Al = 1.0) and its pore size is 4Å. It is stable in wide range of aqueous solvents and does not wash out quickly from membranes [46].

In this study, we hypothesized that: (i) two different nanofillers in PSf polymer matrix could result in the improved dispersion of nanofiller (while any single type of nanofiller could cause agglomeration due to poor dispersion); (ii) one nanofiller may improve the permeability while other may increase the rejection without sacrificing the other one; and (iii) two hydrophilic nanofillers could increase the hydrophilicity of MMMs and fouling resistance as compared to a single hydrophilic filler. As far as we are aware, this is the very first study on the combined use of the two porous fillers (Zeolites-MOFs) in MMMs for any water purification application and especially for the enhancement of antifouling properties of membranes.

## 2. Materials and Methods

### 2.1. Materials

ZrCl_4_ was purchased from MERCK Schuchardt Hamburg, Germany. Terephthalic acid (99+% pure, MW 166.13 g/mol) was purchased from ACROS Organics, Geel, Belgium. Methanol and Chloroform (analytical reagent grade) were bought from Fisher Scientific, Geel, Belgium. PSf polymer and humic acid were purchased from Sigma Aldrich, Hamburg, Germany. NMP (1-methyl-2-pyrrolidinone) and N, N-dimethylformamide (DMF) were obtained from CARLO ERBA Reagents (Le Vaudreuil, France). Zeolite 4A (Molecular Sieve 4A, Powder) was purchased from ACROS Organics, Geel, Belgium.

### 2.2. Preparation of MOF (UiO-66)

UiO-66 was synthesized by following the process described in Anjum et al. [43]. Equimolar Zirconium tetrachloride (6.4 g) and terephthalic acid (4.6 g) were dissolved into 140 mL DMF. The obtained mixture was poured into a Teflon lined autoclave and then heated at 120 °C for 24 h. The synthesized mixture was washed with DMF, Methanol, and Chloroform for 45 min each with centrifugation at 4000 rpm. The crystalline UiO-66 dried in oven at 90 °C for 24 h.

### 2.3. Preparation of Membranes

Phase inversion technique was adopted for membrane fabrication. PSf polymer, Zeolite 4A and UiO-66 nanofillers were dried overnight at 110 °C in an oven. 18 wt% PSf concentrations were taken for all membranes’ casting solutions. 

For the casting of neat PSf membrane, dope solution was prepared by adding 18 wt% PSf pellets in 82 wt% NMP solvent and stirred for 24 h. For the fabrication of single filler MMMs, different amount of nanofillers (Zeolite 4A/UiO-66) were first added in 82 wt% NMP solvent. For the MMMs with combined fillers, equal amounts of both nanofillers were added together in NMP solvent and stirred for 3 h to achieve a homogenous suspension. Then, 18 wt% PSf was added in the mixture in 3 parts and stirred to get a homogenous solution. Air bubbles in casting solutions were removed with the help of sonication. The loadings of nanofiller (Zeolite 4A or/and UiO-66) were selected as 0.5 wt%, 1 wt% and 2 wt%. A non-woven polypropylene/polyethylene fabric (Novatex-2471) used as a support layer was attached with glass plate and wetted by NMP solvent to avoid the penetration of casting solution in the support pores. The polymer film was then casted with a filmograph (K4340 Automatic Film Applicator, Elcometer, Manchester, UK) at pre-set transverse speed of 70 mm per second. The glass plate with thin film on top was immersed in a coagulation bath containing a non-solvent (water), where an exchange of solvent and non-solvent took place. The membranes were air dried followed by drying in the oven for 24 h at 60 °C. Table 1 presents the composition of all membranes prepared with different loadings of nanofillers.

### 2.4. Characterization of UiO-66 and Membranes

Crystalline structure and phase purity of synthesized UiO-66 was recorded by X-ray diffraction (XRD). The sample was analyzed by using Philips made PAN-analytical X’Pert Pro PW3050/60 diffractometer, operating at 45 kV and 40 mA with CuKα radiation. At a wavelength λ = 0.1540 nm, sample analyzed with scan range (2θ) from 5–60°, step size 0.03°and time per step 0.65 s (total counting time 1200 s). To analyze the functional groups in UiO-66, Fourier Transform Infrared (FTIR) analysis was conducted. The samples were analyzed on Thermo-Nicolet 6700 P FTIR Spectrometer (USA), Purging: Helium Gas. The measurements were performed in absorbance. The spectra were obtained with 256 scans with resolution 0–100 nm and scans range (wavelength) 4000–400 cm^−1^. 

The pore characteristics of UiO-66 were analyzed by Brunauer–Emmet–Teller (BET). The degassing temperature was kept 300 °C for 12 h with relative pressure range of 0.000399–0.037083. BET was performed with V-Sorb 2800P instrument of Gold APP Instruments Company. The thermal stability of UiO-66 was assessed using a Thermo Gravimetric Analyzer (TGA) instrument SDT Q600 series with a temperature range of 25–900 °C under a flow of nitrogen gas. The morphology of filler particles as well as MMMs was characterized by Scanning Electron Microscopy (SEM). Analysis was performed by using a Tescan Orsay Holding VEGA-3 LMU, Scanning Electron microscope, Czech Republic. UTHSCSA image tool was used. Cross-sectional pieces of membrane were prepared by freezing the samples in liquid nitrogen. 

Water contact angle (CA) of membranes was calculated by drop shape analyzer (KRÜSS instruments, Model: DSA30, Hamburg, Germany). Water droplet was dropped on the membrane surface and static contact angle was calculated immediately. Contact angle was measured at five different locations of each sample and the average values were used. Equilibrium Water Content (EWC) of membranes is directly associated with the porosity of membranes. It also indicates the property of hydrophilicity of membranes. The membrane samples were cut in square shape with area of 4 cm^2^ and then immersed in deionized water for 24 h. The samples were weighed immediately after removing the excess water and dried for 48 h at 80 °C in an oven. The dry membranes then weighed again. The EWC% was calculated by Equation (1):

EWC (%) = ((W_w_ − W_d_))/W_w_ × 100 (1)

where W_w_ is weight of wet membrane and W_d_ is the weight of dry membrane.

### 2.5. Membrane Performance

#### 2.5.1. Permeation and Rejection

**Figure A1:**
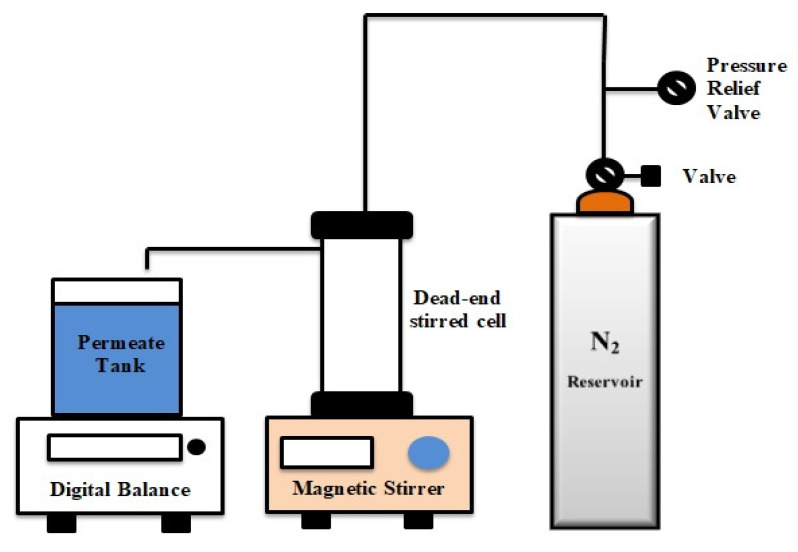
Schematic diagram of dead-end filtration experimental setup.

Dead-end filtration cell (STERLITECH Corporation, Kent, WA, USA, HP4750 Stirred Cell) was used to evaluate the performance of neat and modified membranes. The filtration cell had processing volume of 300 mL with an active membrane area of 14.6 cm^2^. The cell was connected to a N_2_ cylinder. All filtration experiments were carried out at room temperature (25 ± 2 °C). The operating pressure was kept at 2 bar. The cell was placed on magnetic stirring plate for stirring the feed solution at speed of 5000 rpm as illustrated in Figure A1 (Appendix A).

Prior compaction of membranes was performed at 2 bar pressure for 30 min until the steady state flux was achieved. Pure water Flux (PWF) was measured using formula described in Equation (2):

J_o_ = (V/(A × ∆t)) (2)

where J_0_ is PWF (Lm^−2^h^−1^), V is volume of permeate (L), A is effective membrane area (m^2^) and Δt is permeation time (h).

Humic acid solution of 2 g/L prepared in deionized water was forced through membranes at same operating pressure of 2 bar. Humic acid rejection ratio was measured by following Equation (3):

R% = (1 − C_p_/C_f_) × 100 (3)

where C_f_ and C_p_ are the concentrations of humic acid in feed and permeate (mg/L), respectively which was measured with UV-VIS spectrophotometer on 254 nm wavelength.

#### 2.5.2. Antifouling Properties of Membranes

##### Static Humic Acid Adsorption

Static humic acid adsorption test was carried out to investigate the fouling behavior of neat and modified membranes. Membranes were cut into a circle shape with 25 mm diameter (area = 49.1 cm^2^). The membranes were dipped in glass vials containing 10 mL of 2 g/L humic acid solution. The vials were agitated with a shaker for 24 h to achieve adsorption equilibrium at room temperature (25 °C). The concentration of humic acid in solution before (C_0_) and after (C) adsorption were measured with UV-VIS spectrophotometer on 254 nm wavelength [47]. The apparent amounts of adsorbed humic acid were calculated using Equation (4)

Q = ((C_0_ − C)/A) × 100% (4)

where Q is the adsorbed amount of humic acid (µgcm^−2^), A is the membrane area (cm^2^), and C_0_ and C are the concentration of humic acid (µg) before and after adsorption, respectively.

##### Flux Recovery Ratio (FRR)

Fouling resistance performance of membranes was analyzed by dynamic humic acid filtration experiments. 2 g/L humic acid aqueous solution was filtered by dead-end filtration experimental setup and humic acid flux was recorded. The flux reduction was characterized by measuring decrease of permeate volume with filtration time. Then fouled membranes were taken out of filtration assembly and washed with deionized water for 30 min with vigorous stirring. Then water flux of washed membrane was measured. Flux recovery ratio (FRR) is used to estimate the antifouling ability of membranes, which was calculated using the following equations [37]:

FRR (%) = [J_2_/J_0_] × 100 (5)

where J_0_ is PWF before fouling and J_2_ is water flux of clean membrane after fouling.

##### Reversible and Irreversible Fouling

To study the antifouling property of membranes, total fouling ratio (F_t_), Reversible fouling ratio (F_r_) and Irreversible fouling ratio (F_ir_) were used [11]. F_t_ is the degree of total flux loss caused by total fouling. A high value of Ft represents a large reduction in flux. The total fouling ratio (Ft) was calculated as follows:

F_t_ (%) = (1 − J_1_/J_0_) × 100 (6)

To distinguish reversible fouling and irreversible fouling, two ratios: F_r_ and F_ir_ were used. F_r_ is calculated by Equation (7) that is the degree of reversible flux loss caused by reversible fouling which can be removed by hydraulic washing.

F_r_ (%) = ((J_2_ − J_1_)/J_0_) × 100 (7)

Fir is defined by Equation (8), which is the degree of irreversible flux loss caused by irreversible fouling that cannot be avoided by hydraulic washing.

F_ir_ (%) = ((J_0_ − J_2_)/J_0_) × 100 (8)

Thus, F_t_ is the sum of F_r_ and F_ir_. Here, J_1_ is humic acid permeate flux, J_0_ is the PWF before fouling and J_2_ is water flux of clean membrane after fouling.

## 3. Results and Discussion

### 3.1. Characterization of UiO-66

**Figure A2:**
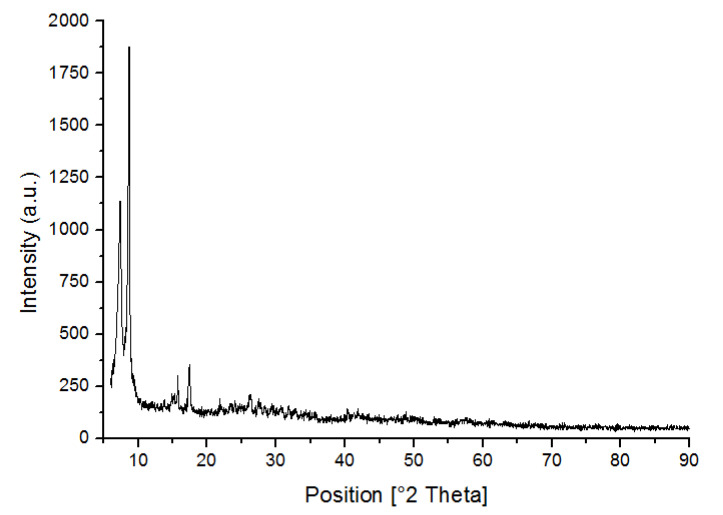
XRD patterns of UiO-66 particles from 5° to 90°.

**Figure A3:**
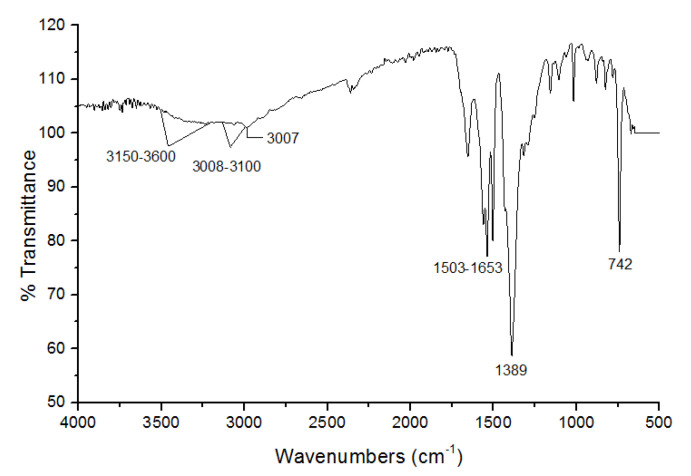
FTIR Spectra of UiO-66 particles in range of 4000–500 cm^−1.^

XRD analyses were performed for the verification of phase purity and crystalline structure of synthesized UiO-66. Figure A2 (Appendix A) shows two high peaks at position (2θ) 7° and 9° corresponding to the planes of face-centered cubic crystal of UiO-66. All the diffraction peaks of the synthesized MOF showed highly crystalline structure and are consistent with UiO-66′s simulated standard patterns [39]. An FTIR spectrum of UiO-66 is represented in Figure A3 (Appendix A), the band at 742 cm^−1^ and 1389 cm^−1^ are assigned as the C–H Stretch and C–O stretch respectively. Bands from 1503–1653 cm^−1^ represent the C=C stretch within the MOF. The band at 3007 cm^−1^ is attributed to the O–H Stretch (uncoordinated COOH). Band between 3008 cm^−1^ and 3100 cm^−1^ represents the aromatic C-H Stretch while the bands between 3150–3600 cm^−1^ are attributed to O–H Stretch within UiO-66 (C_48_H_28_O_32_Zr_6_). All these functional groups endow UiO-66 MOF’s high hydrophilic characteristic. Therefore, UiO-66 is expected to be able to improve the hydrophilicity of the membrane after blending in polymer matrix. These bands are in line with the literature [48].

**Figure A4:**
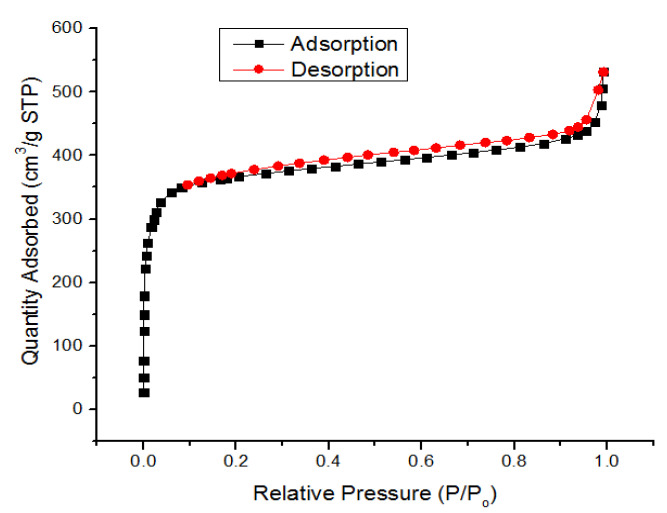
Nitrogen isotherm sorption plot of UiO-66.

**Figure A5:**
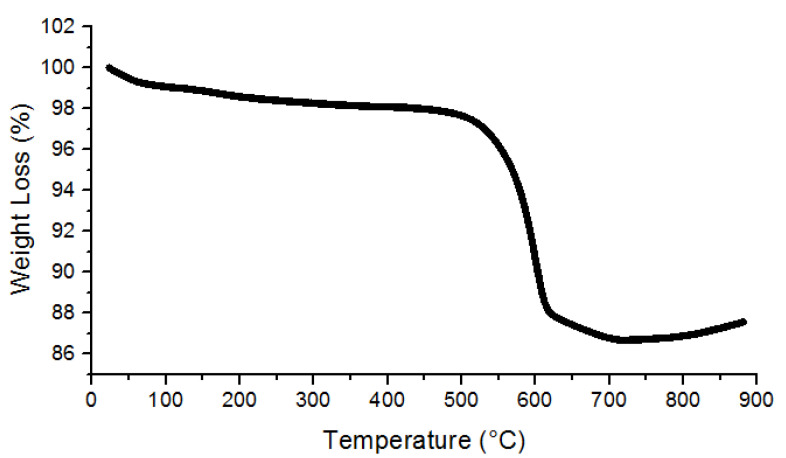
TGA curve of UiO-66 particles.

The surface area of UiO-66 reported from BET measurements is 1476 m^2^/g indicating the high porosity of MOF. Its micro-pore volume is 0.509 cm^3^/g calculated by t-plot method. The nitrogen adsorption and desorption isotherms are shown in Figure A4. UiO-66 sample showed characteristic Type I sorption isotherms, indicating typical microporous materials. TGA results are presented in Figure A5 (Appendix A), indicating that UiO-66 is thermal stable up to 500 °C. UiO-66 started weight loss of 1% up to 150 °C which ascribes desorption of water and trapped residual solvent i.e., DMF in UiO-66 framework. TGA plateau from 160–500 °C indicates the weight loss of about 1% which acknowledges the de-hydroxylation of UiO-66 framework. H_2_O removed from Zr−O metal clusters which were present in the form of –OH groups. Residual solvent and water removed simultaneously which was coordinating with Zr-metal to compensate the linker deficiencies. Temperature range from 510–600 °C shows the significant weight loss of about 10% indicating the complete burning of framework present in UiO-66. After that, continuous degradation is observed till 900 °C. About 88% residual material at 900 °C indicated that the prepared UiO-66 proved to be more stable as compared to what is reported in the literature [49] where almost 40% of the material remained at 800 °C.

**Figure A6:**
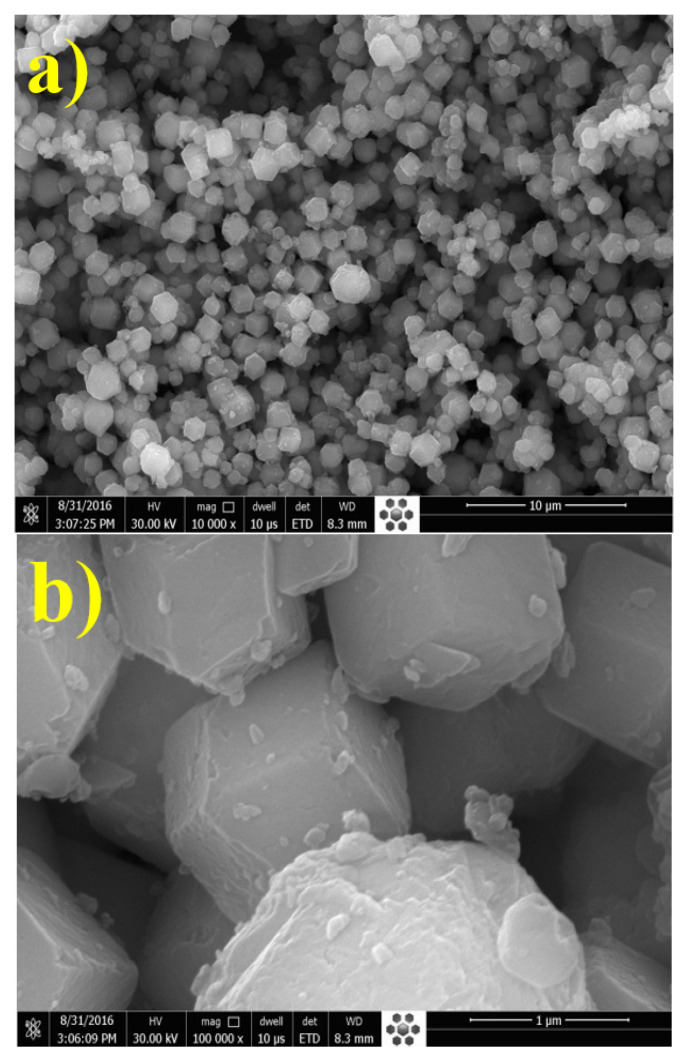
SEM morphology of the as-synthesized MOF UiO-66 particles: (**a**) morphology with 10,000× magnification, (**b**) a zoom-in observation of UiO-66 morphology with 100,000× magnification.

SEM images showed the crystalline morphology of synthesized MOF as shown in Figure A6a,b (Appendix A). UiO-66 particles possess cubic shape with sharp edges. The cubic crystalline structure of UiO-66 is verified with the existing literature [43]. The average diameter of each nanofiller particle is around 600 nm with standard deviation of 100 nm. This is also consistent with the values reported in the existing literature [44].

### 3.2. Characterization of Membranes

#### 3.2.1. Morphology of MMMs

**Figure 1:**
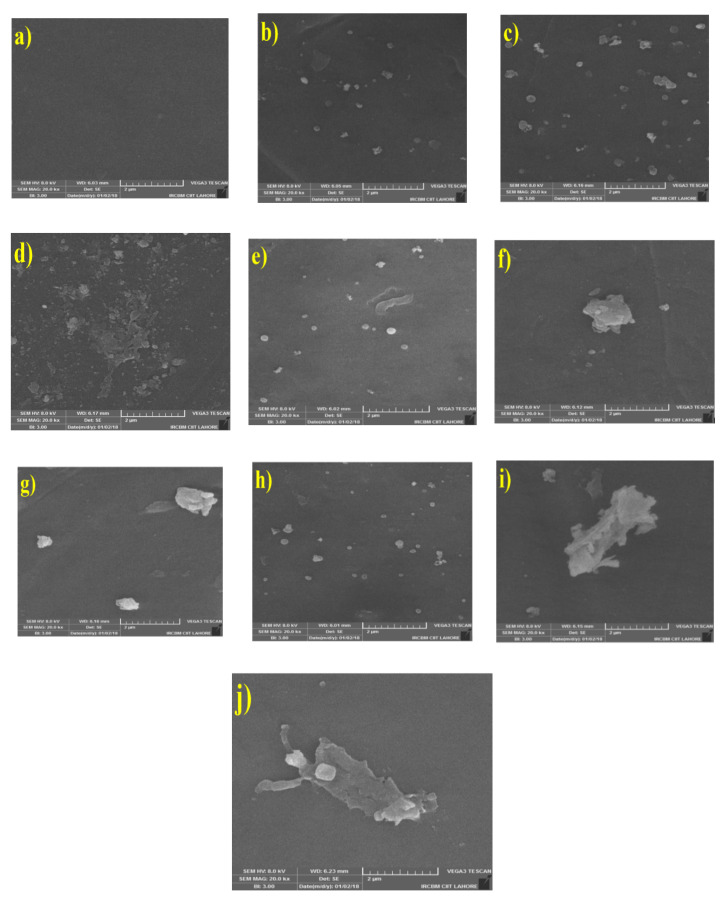
Surface SEM images of membranes (**a**) Neat PSf membrane; (**b**–**d**) PSf/Zeolite 4A membranes; (**e**–**g**) PSf/UiO-66 membranes; (**h**–**j**) PSf/Zeolite 4A-UiO-66 membranes with different nanofiller loadings (0.5 wt%, 1 wt%, 2 wt%, respectively).

**Figure 2:**
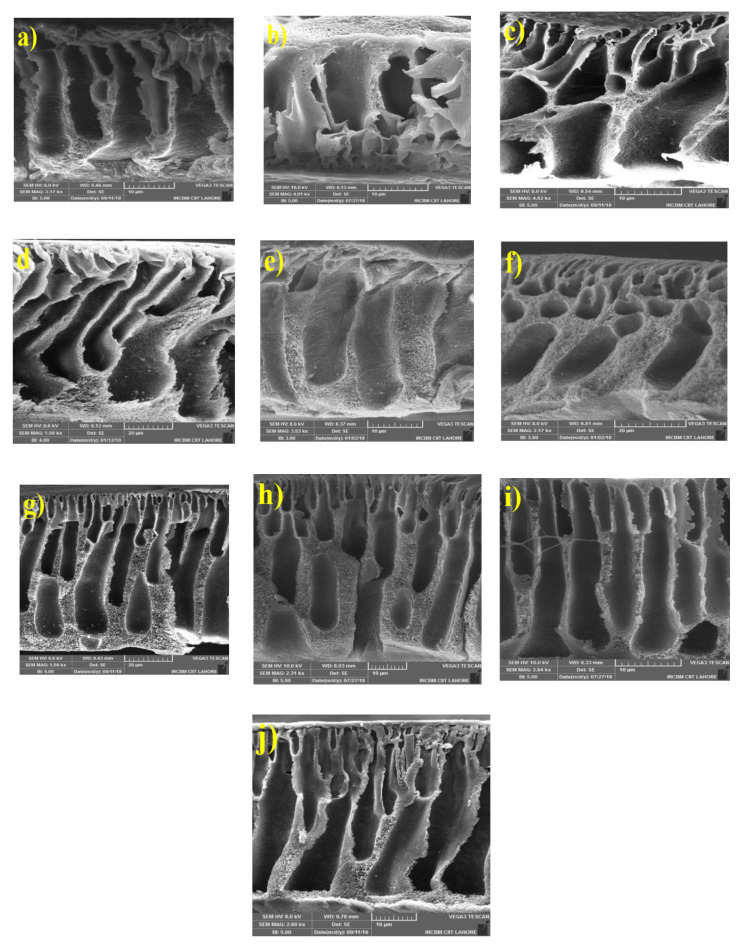
Cross section SEM images of membranes (**a**) Neat PSf membrane; (**b**–**d**) PSf/Zeolite 4A membranes; (**e**–**g**) PSf/UiO-66 membranes; (**h**–**j**) PSf/Zeolite 4A-UiO-66 membranes with different nanofiller loadings (0.5 wt%, 1 wt%, 2 wt%, respectively).

Surface morphology and cross section of neat PSf and MMMs filled with different nanofiller loadings (0.5 wt%, 1 wt% and 2 wt%) of Zeolite 4A, UiO-66, and their combination are shown in Figure 1 and Figure 2 respectively. Neat PSf membrane (Figure 1a) has uniform and smooth surface. Figure 1b–d shows the surface morphology of PSf/Zeolite 4A membranes. As the concentration of Zeolite 4A nanofiller is increased, the amount of nanofillers on the surface of membranes also increased. Figure 1e–g,h–j shows the surface morphology of PSf/UiO-66 and PSf/Zeolite 4A-UiO-66 membranes, respectively. Both PSf/UiO-66 (0.5 wt%) and PSf/Zeolite 4A-UiO-66 (0.5 wt%) membranes exhibited uniform dispersion of nanofiller on the surface of membranes. PSf/UiO-66 and PSf/Zeolite 4A-UiO-66 membranes with nanofiller loadings (1 wt% and 2 wt%) possessed nanofiller agglomerates on the membrane surface that can lead to pore clogging of membrane pores. This agglomeration cannot be seen in PSf/Zeolite 4A membranes. It is an accepted hypothesis that nanoparticles which own high surface areas have high tendency to form agglomerates [1] and UiO-66 nanofiller possesses much higher surface area (1476 m^2^/g) than Zeolite 4A nanofillers (369 m^2^/g) [50]. PSf/Zeolite 4A-UiO-66 membranes showed larger agglomerates as compared to single filler PSf/UiO-66 membranes due to the interaction of two nanofillers of different nature.

Figure 2a–j shows the cross section of neat PSf and MMMs with different nanofillers: Zeolite 4A (b–d), UiO-66 (e–g) and Zeolite 4A-UiO-66 (h–j). All fabricated membranes exhibited similar porous and asymmetrical structure due to the exchange of solvent and non-solvent in phase inversion process [51]. In all MMMs, macrovoids become larger with the increased nanofiller loading in polymer matrix due to rapid exchange of solvent and non-solvent in the diffusion of water from coagulation bath to the casting polymer film which is strongly facilitated by hydrophilic nanofillers [4]. For all MMMs, it is observed that 0.5 wt% and 1 wt% nanofiller loaded MMMs possess smaller macrovoids while for MMMs with 2 wt% loading of nanofillers, larger finger-like macrovoids are observed that are also more in numbers. These wider pores provide new pathways for water molecules and increase the water permeability of membranes.

#### 3.2.2. Static Water Contact Angle

**Figure 3:**
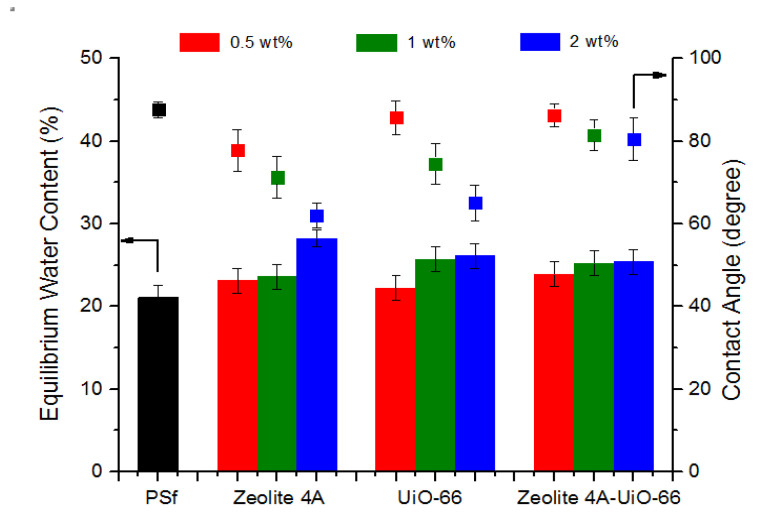
Hydrophilicity parameters of neat PSf and MMMs.

Hydrophilicity of membranes was determined through static water contact angle. Figure 3 represents the water contact angle of neat PSf membrane, PSf/Zeolite 4A, PSf/UiO-66 and PSf/Zeolite 4A-UiO-66 blended membranes with different loadings (0.5 wt%, 1 wt% and 2 wt%). The results show that neat PSf membrane possesses highest value of contact angle (88°) which represents its lowest hydrophilicity as compared to all MMMs. Lowest hydrophilicity of neat PSf membrane is due to the intrinsic hydrophobic property of PSf polymer. The results of MMMs showed the decreasing trend of contact angle with increased loading of nanofillers in casting solution from 0.5 wt% to 2 wt%. This indicates that hydrophilicity of membrane has increased due to the presence of hydroxyl groups on membrane surface which are provided by nanofillers [17]. Higher surface hydrophilicity of membrane is favorable for PWF, resistance towards humic acid adsorption, and fouling.

Overall, PSf/Zeolite 4A membranes with all nanofiller loadings exhibited higher hydrophilicity than PSf/UiO-66 and PSf/Zeolite 4A-UiO-66 membranes. This could be expected due as Zeolite 4A consists of high aluminum content in Zeolite framework as its Si/Al ratio is 1 which makes it highly hydrophilic [52]. Second, Zeolite 4A has resilience towards agglomeration due to the lower surface area. PSf/UiO-66 membranes also exhibited higher hydrophilicity than the neat PSf membrane. However, it showed less hydrophilicity than PSf/Zeolite 4A membranes. This could be due to the less hydrophilic character of UiO-66 in comparison to Zeolite 4A. 

PSf/Zeolite 4A-UiO-66 membranes showed the least hydrophilicity which could be attributed to the agglomeration tendency of two nanofillers of different nature as these membranes showed larger agglomerates on the membrane surface (Figure 1), which could reduce the effective surface of nanoparticles and therefore, reduce the hydroxyl groups on the surface of MMMs [1].

#### 3.2.3. Equilibrium Water Content (EWC)

Equation (1) is used to measure the EWC of membranes which is directly related to porosity of membranes. EWC of neat PSf membrane, PSf/Zeolite 4A, PSf/UiO-66 and PSf/Zeolite 4A-UiO-66 membranes is illustrated in Figure 3. The results indicate that PSf membrane showed the least value of EWC. All MMMs exhibited higher EWC with increased nanofiller loading from 0.5 wt% to 2 wt%. This could be due to the formation of more and larger macrovoids (see Figure 2) and more hydrophilicity of membranes (see Figure 3) that helps to attain more water molecules in the pores of membranes [44]. At low nanofiller loading (0.5 wt%), PSf/Zeolite 4A-UiO-66 membrane showed high EWC as compared to PSf/Zeolite 4A and PSf/UiO-66 membranes. Overall, PSf/Zeolite 4A membranes showed higher EWC as compared to PSf/UiO-66 and PSf/Zeolite 4A-UiO-66 membranes as PSf/Zeolite 4A membranes exhibited superior hydrophilic property as discussed earlier.

### 3.3. Membrane Performance

#### 3.3.1. Permeation and Rejection

**Figure 4:**
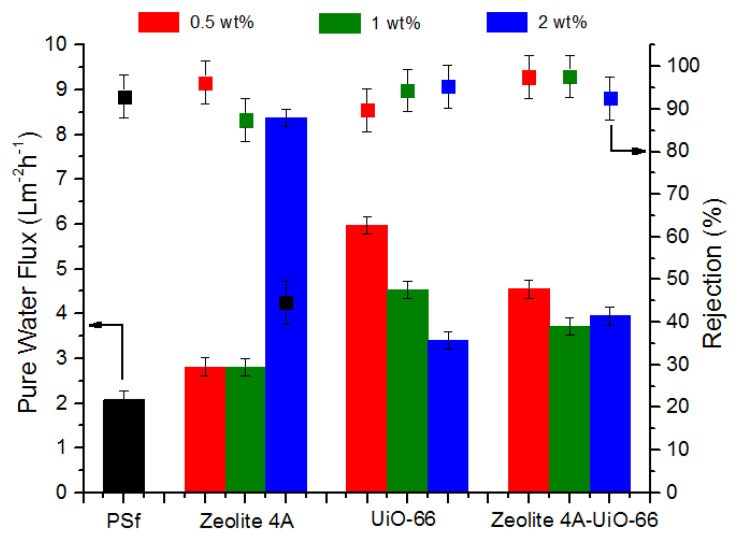
Pure Water Flux and humic acid rejection of neat PSf and MMMs.

PWF of neat PSf membrane and MMMs was determined by Equation (2). PWF was calculated at trans-membrane pressure of 2 bar for 300 min. The obtained results are presented in Figure 4 which indicates that all MMMs showed higher PWF (J_0_) than neat PSf membrane. PSf/Zeolite 4A (0.5 wt%) and (1 wt%) membrane showed around 33% higher PWF than neat PSf membrane. As the loading of Zeolite 4A nanofiller is increased up to 2 wt%, this difference of PWF increased from 33% to 300% in comparison to neat PSf membrane. This increased water flux could be attributed to three different factors. First, with the addition of nanofiller, larger macrovoids in the membrane layer formed as shown in SEM images (Figure 2), which lessens the resistance to water molecules and provides more water transport ways to enhance the permeation of water, and resulted in high water flux. Second, as the hydrophilicity of membranes increased with high nanofiller loading, the hydrophilic membrane attracted more water molecules as confirmed by EWC (see Figure 3) and increased the water transportation in membrane. In addition, third, PSf/Zeolite 4A membranes have resilience towards agglomeration (see Figure 1) so probably membrane pores remained open for water permeation unlike other MMMs.

PSf/UiO-66 and PSf/Zeolite 4A-UiO-66 (0.5 wt%) membranes exhibited 186% and 119% more PWF respectively as compared to neat PSf membrane. With the increase of nanofiller loading from 0.5 wt% to 2 wt%, the PWF of both membranes is decreased significantly but remained higher than neat PSf membrane. These membranes showed the opposite trend of PWF as compared to PSf/Zeolite 4A membranes. Pore size, hydrophilicity, and EWC of membranes increased with increased loading of nanofiller (see Figure 2 and Figure 3) but these characteristics of membrane seem to be dominated by the agglomeration tendency of nanofillers despite the fact that sonication of casting solution was executed to thoroughly disperse the nanofillers in polymer solution. Agglomeration on the surface of membranes increased with increased nanofiller loading as shown in SEM images (Figure 1) which may block the pores on membrane surface and could result in the resistance for water transport and caused low water flux. 

The separation performance of neat PSf and MMMs was evaluated using 2 g/L humic acid aqueous solution with 2 bar operating pressure. The humic acid fluxes of all membranes declined until they get steady state. The rejection of membranes was measured using Equation (3). The humic acid rejection results of neat PSf and MMMs are shown in Figure 4. Neat PSf membrane showed rejection of about 98%. PSf/Zeolite 4A (0.5 wt%) membrane showed 96% humic acid rejection. As the loading of Zeolite 4A nanofiller increased from 0.5 wt% to 2 wt%, rejection decreased from 96% to 45%, which can be mainly due to the formation of larger macrovoids in the membrane layer (see Figure 2), which allow the humic acid particles to pass through the pores and lessen the rejection [53]. 

PSf/UiO-66 membranes showed the opposite trend of humic acid rejection as compared to PSf/Zeolite 4A membranes. PSf/UiO-66 (0.5 wt%) membrane presented 90% rejection less than neat PSf membrane. Humic acid rejection increased from 90% to 95% as UiO-66 nanofiller loading increased from 0.5 wt% to 2 wt% in casting solution. This can be caused by two properties of membranes. First, it is witnessed from SEM images that UiO-66 membranes have affinity of agglomeration with high nanofiller loadings and second, membranes possess wider pores with more nanofiller concentration. So these agglomerates may block the pores on membrane surface and did not allow the humic acid to pass through them and result in more rejection [51]. 

PSf/Zeolite 4A-UiO-66 (0.5 wt%) and (1 wt%) membranes showed humic acid rejection of 97% and 99% respectively. PSf/Zeolite 4A-UiO-66 (1 wt%) membrane showed highest rejection among all MMMs and neat membrane. The addition of these two nanofillers (Zeolite 4A and UiO-66) improved the membranes’ surface hydrophilicity which led to a lower interaction and affinity between membrane surface and humic acid and resulted in better rejection. As the loading of nanofiller is further increased to 2 wt%, the rejection is decreased to 92% because macrovoids in the membrane layer become larger (see Figure 2), which allows humic acid to pass through them [15].

Single filler MMMs (PSf/Zeolite 4A and PSf/UiO-66) showed a typical tradeoff between permeability and rejection. In PSf/Zeolite 4A membranes, PWF increased with high nanofiller loading from 0.5 wt% to 2 wt%, while its rejection decreased significantly. PSf/UiO-66 membranes showed the opposite trend as compared to PSf/Zeolite 4A membranes. PSf/UiO-66 membranes exhibited lower PWF with high nanofiller loading while its humic acid rejection improved but it remained less than neat PSf membrane. PSf/Zeolite 4A-UiO-66 membranes did not show this tradeoff. PSf/Zeolite 4A-UiO-66 membranes with all three loadings showed better PWF than neat PSf membrane and PSf/Zeolite 4A-UiO-66 (0.5 wt%) and (1 wt%) membranes exhibited high rejection of 97% and 99% respectively. 

#### 3.3.2. Antifouling Properties of Membranes

##### Static Humic Acid Adsorption

**Figure 5:**
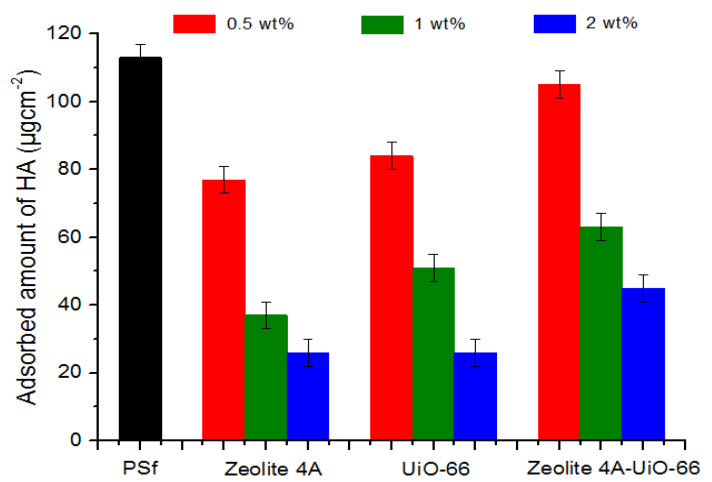
Adsorbed amount of humic acid on neat PSf and MMMs.

The antifouling behavior of prepared membranes was evaluated by determining the amount of humic acid adsorbed on the membrane surface through the formula described in Equation (4). The results of adsorbed humic acid on neat PSf membrane, PSf/Zeolite 4A, PSf/UiO-66 and PSf/Zeolite 4A-UiO-6 membranes are shown in Figure 5. The results indicate that neat PSf membrane shows highest value of static humic acid adsorption of 113 µgcm^−2^ which was due to the inherent hydrophobicity of PSf which make it prone to the adsorption of organic matter. 

In all MMMs, as the nanofiller loading increased from 0.5 wt% to 2 wt% in casting solution, humic acid adsorption decreased. This is due to the increased hydrophilic behavior of prepared membranes and their attraction towards water. As the loading of nanofiller increases, more hydroxyl groups become available to make a bond with water, so as a result a hydration layer is formed on the membrane surface which prevents the direct contact of humic acid on the membrane surface and thus, reduces humic acid adsorption [54]. Overall, all membranes showed a similar trend just like hydrophilicity of membranes (see Figure 3). Membranes which possessed higher hydrophilicity, also exhibited less affinity for humic acid adsorption and vice versa.

##### Flux Recovery Ratio (FRR)

**Figure 6:**
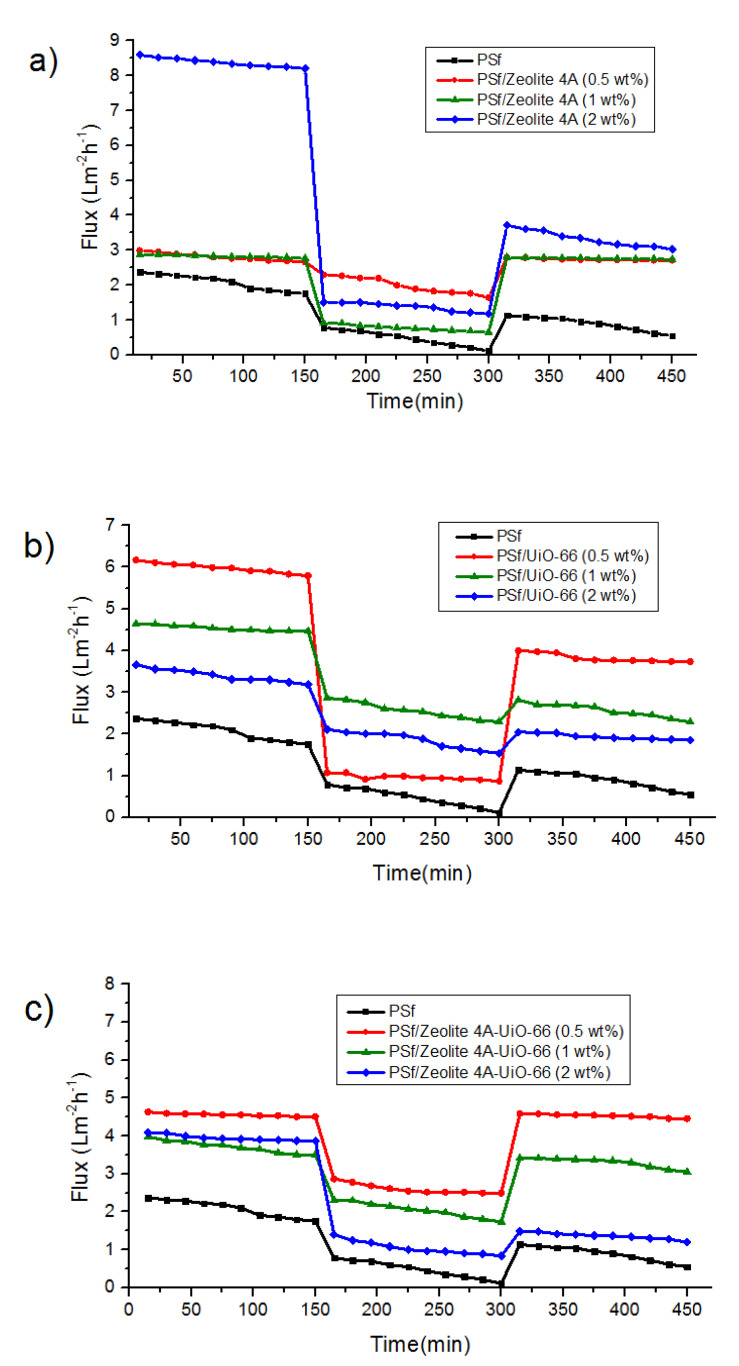
Flux versus time for mixed-matrix PSf membranes with different nanofiller concentrations during three steps: water flux for 150 min, humic acid solution flux for 150 min, and water flux for 150 min after 30 min washing with distilled water (**a**) PSf/Zeolite 4A, (**b**) PSf/UiO-66 and (**c**) PSf/Zeolite 4A-UiO-66.

The performance of neat PSf membrane and all MMMs was characterized by measuring fluxes for three different time periods as shown in Figure 6. Before measuring these fluxes, membranes were compacted for 30 min at 2 bar pressure to get a steady flux. First 150 min span shows PWF, when pure water is passed through membranes. Next 150 min show humic acid flux when in dead-end filtration cell deionized water is replaced with humic acid aqueous solution. Because of humic acid fouling, water flux of membranes rapidly reduced. Humic acid molecules may deposit on the membrane surface and causes a sharp drop in flux at the first few minutes of operation. The reduced flux is recorded. After fouling by humic acid, the membranes were washed with deionized water for 30 min to attain the flux recoveries of membranes. Last 150 min show the water flux of all membranes after washing the fouled membranes.

In all MMMs, both PWF before humic acid filtration and after washing of fouled membrane improved by increasing the nanofiller content as compared to neat PSf membrane due to the high hydrophilicity of MMMs. In case of PSf/Zeolite 4A membranes, the higher water fluxes were observed for 2 wt% blend membrane and these results showed consistency with hydrophilicity of membranes (see Figure 3) In case of PSf/UiO-66 and PSf/Zeolite 4A-UiO-66 membranes, 0.5 wt% membranes showed highest water fluxes because of the uniform dispersion of nanofillers on the membrane surface (Figure 1). 

**Figure 7:**
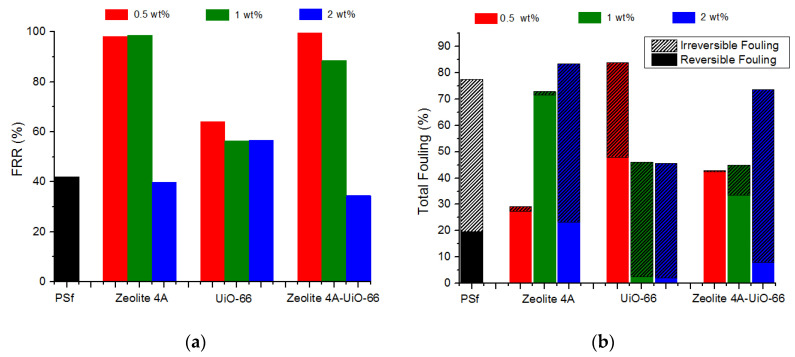
Fouling parameters of neat PSf and MMMs. (**a**) Flux Recovery Ratio of neat PSf and MMMs (**b**) Reversible, Irreversible and Total fouling of neat PSf and MMMs.

For better understanding of the antifouling performance of membranes, FRR was calculated using Equation (5). The higher value of FRR represents the better antifouling property of membrane. The detailed FRR of neat PSf and MMMs is represented in Figure 7a. The results illustrated that neat PSf membrane when washed with deionized water attained 42% flux recovery indicating poor antifouling property. However, when Zeolite 4A and PSf/Zeolite 4A-UiO-66 (0.5 wt%) nanofillers are added in PSf polymer matrix, FRR reached up to 98% and 99% respectively. Membranes at low nanofiller content possess better FRR; however, at higher nanofiller content, the FRR slightly decreased to 39% and 34% respectively implying that severe humic acid fouling occurred caused by larger macrovoids in the membrane layer (Figure 2), which allows the humic acid to enter the pores. 

PSf/UiO-66 (0.5 wt%) membrane possess FRR of 64% higher than neat PSf membrane due to the introduction of hydrophilic groups on the membrane surface and more water adsorption capacity of nanofillers due to their large surface area [11] which resulted in reduced fouling. However, FRR slightly decreased to 57% for PSf/UiO-66 (2 wt%) due to severe fouling by humic acid, which was because of larger macrovoids and more agglomeration on the membrane surface, which decreased the surface area of the hydrophilic group of nanofiller and possibly increased the deposition of humic acid on/in membrane pores. Along with FRR, Reversible and Irreversible fouling ratios were used to further study the antifouling property of membranes.

##### Reversible and Irreversible Fouling

Membrane fouling occurs by the adsorption of foulant material on the membrane surface or their entrapment inside the pores. Membrane fouling consists of reversible and irreversible fouling. Reversible fouling is adsorption of foulant material loosely on the surface of membrane which is easily removed by hydraulic washing. However, irreversible fouling is the strong attachment of foulant material on the membrane surface and inside the pores of membrane. High value of irreversible fouling ratio indicates the poor antifouling behavior of membranes [55]. 

Figure 7b shows total fouling ratio (Ft), reversible fouling ratio (Fr), and irreversible fouling ratio (Fir) of neat PSf and all MMMs calculated by Equations (6)–(8) respectively by estimating water flux before humic acid fouling and after hydraulic cleaning to compare the antifouling properties of membranes. The results show that the neat PSf membrane exhibited 77.6% total fouling ratio with high irreversible fouling ratio as it possesses intrinsic hydrophobicity. When 0.5 wt% Zeolite 4A nanofiller is added in PSf matrix, the total fouling ratio is significantly reduced to 29% with lower irreversible fouling ratio indicating better antifouling property. With high Zeolite 4A loading (2 wt%), the total fouling ratio is increased to 84% along with increased irreversible fouling ratio which was due to the formation of more macrovoids in the membrane layer (see Figure 2) that entrapped the humic acid particles in the pores of the membrane and resulted in severe irreversible fouling. 

PSf/UiO-66 membranes followed the opposite trend as compared to PSf/Zeolite 4A membranes. In PSf/UiO-66 membranes, as the nanofiller loading is increased, total fouling ratio has decreased significantly with very low reversible fouling and slightly high irreversible fouling ratios because of agglomeration of UiO-66 nanofiller at higher concentration (see Figure 1). PSf/Zeolite 4A-UiO-66 (0.5 wt%) membrane showed 42.8% total fouling with least irreversible fouling among all fabricated membranes indicating best antifouling properties. Furthermore, adding nanofillers at higher loadings (1 wt% and 2 wt%) resulted in total fouling ratio of 45% and 74% respectively with high irreversible fouling ratio due to the agglomeration of nanofillers in the polymer matrix. 

## 4. Conclusions

In this study, MMMs were successfully prepared via phase inversion technique by incorporating Zeolite 4A, UiO-66 and their combination in a PSf matrix with three different loadings (0.5 wt%, 1 wt% and 2 wt%). Addition of hydrophilic nanofillers caused significant changes in the morphology and chemistry of membranes. High nanofiller loading in polymer matrix resulted in agglomeration of nanofiller on the membrane surface of PSf/UiO-66 and PSf/Zeolite 4A-UiO-66 membranes. These hydrophilic nanofillers improved the macrovoid structure and increased the hydrophilicity of all MMMs which resulted in higher PWF as compared to neat PSf membrane. However, PSf/Zeolite 4A and PSf/UiO-66 membranes showed the opposite trend of PWF and humic acid rejection with increased nanofiller loadings from 0.5 wt% to 2 wt% due to the presence of agglomeration on membrane surface (PSf/UiO-66) and wide macrovoids in membrane matrix. This typical tradeoff between permeability and selectivity was not present in membranes with combined fillers (PSf/Zeolite 4A-UiO-66). All the MMMs at low nanofiller loadings (0.5 wt% and 1 wt%) showed good antifouling properties as compared to neat PSf membrane. PSf/Zeolite 4A-UiO-66 (0.5 wt%) membrane showed superior antifouling properties without sacrificing the permeability and selectivity. PSf/Zeolite 4A-UiO-66 (0.5 wt%) membrane showed superior antifouling properties without sacrificing the permeability and selectivity. However, contrary to our hypothesis, PSf/Zeolite 4A-UiO-66 membranes with higher nanofiller loadings are not showing promising results (in terms of nanofiller dispersion, hydrophilicity, EWC and humic acid adsorption) as compared to single filler membranes due to nanofiller agglomeration as shown in SEM results which may be majorly due to the compatibility issues between two types of nanofillers of different nature with their own transport channels.

## Figures and Tables

**Table 1:** Composition of membranes.

Membrane	Nanofiller Composition	
	Zeolite 4A (wt%)	UiO-66 (wt%)
Neat PSf	-	-
Zeolite 4A (0.5 wt%)	0.5	-
Zeolite 4A (1 wt%)	1.0	-
Zeolite 4A (2 wt%)	2.0	-
UiO-66 (0.5 wt%)	-	0.5
UiO-66 (1 wt%)	-	1.0
UiO-66 (2 wt%)	-	2.0
Zeolite 4A-UiO-66 (0.5 wt%)	0.25	0.25
Zeolite 4A-UiO-66 (1 wt%)	0.5	0.5
Zeolite 4A-UiO-66 (2 wt%)	1.0	1.0

## Appendix A

Dead-end filtration experimental setup

XRD patterns of UiO-66 nanofiller

FTIR Spectra of UiO-66 nanofiller

Sorption Isotherms of UiO-66 nanofiller

TGA curve of UiO-66 nanofiller

SEM morphology of UiO-66 nanofiller
